# Efficiency and Toxicity of Ruxolitinib as a Salvage Treatment for Steroid-Refractory Chronic Graft-Versus-Host Disease

**DOI:** 10.3389/fimmu.2021.673636

**Published:** 2021-06-30

**Authors:** Dong Wang, Yin Liu, Xiaoxuan Lai, Jia Chen, Qiao Cheng, Xiao Ma, Zhihong Lin, Depei Wu, Yang Xu

**Affiliations:** ^1^ National Clinical Research Center for Hematologic Diseases, Jiangsu Institute of Hematology, Key Laboratory of Thrombosis and Hemostasis of Ministry of Health, The First Affiliated Hospital of Soochow University, Suzhou, China; ^2^ Institute of Blood and Marrow Transplantation, Collaborative Innovation Center of Hematology, Soochow University, Suzhou, China; ^3^ Department of Hematology, Soochow Hopes Hematonosis Hospital, Suzhou, China; ^4^ Soochow Yongding Hospital, Department of Affiliated Renji Hospital of Shanghai Jiao Tong University of Medicine, Suzhou, China

**Keywords:** hematopoietic stem cell transplant, ruxolitinib, steroid-refractory chronic graft-versus-host disease, overall response rate, overall survival

## Abstract

Graft-versus-host disease (GVHD), especially steroid-refractory GVHD, remains a life-threatening complication after hematopoietic stem cell transplantation (HSCT). The effect of the JAK1/2 kinase inhibitor ruxolitinib on treating steroid-refractory acute GVHD has been verified by the REACH1/2 study; however, its safety and efficacy in patients with steroid-refractory chronic GVHD (SR-cGVHD) remain unclear. In this retrospective study, 70 patients received ruxolitinib as a salvage therapy for SR-cGVHD. Twenty-four weeks after ruxolitinib treatment, the overall response rate (ORR) was 74.3% (52/70), including 34 patients who achieved complete remission (CR) and 18 who achieved partial remission (PR). The main adverse event was cytopenia, which occurred in 51.4% (36/70) of patients. After ruxolitinib treatment, the percentage of CD4 cells increased from 18.20% to 23.22% (*P*<0.001), while the percentages of NK (CD16^+^CD56^+^) cells and regulatory T cells (CD4^+^CD127 ^±^ CD25^+^) decreased (*P*<0.001, *P*<0.001). Among the B cell subsets, the proportion of total B cells approximately tripled from 3.69% to 11.16% (*P*<0.001). Moreover, we observed a significant increase in IL-10 levels after ruxolitinib treatment (*P*=0.025) and a remarkable decrease in levels of suppression of tumorigenicity 2 (ST2) from 229.90 ng/ml to 72.65 ng/ml. The median follow-up after the initiation of ruxolitinib treatment was 401 (6-1076) days. The estimated one-year overall survival rate of the whole group was 66.0% (54.4–77.6%, 95% CI), and the one-year overall survival rate of patients with mild and moderate cGVHD was 69.6% (57.4–81.8%, 95% CI), which was better than that of patients with severe cGVHD (31.3%, 0.0–66.2%, 95% CI) (*P*=0.002). Patients who achieved a CR and PR achieved better survival outcomes (84.5%, 73.9–95.1%, 95% CI) than those who showed NR to ruxolitinib treatments (16.7%, 0–34.3%, 95% CI) (*P*<0.001). At the final follow-up, cGVHD relapse occurred in six patients after they reduced or continued their ruxolitinib doses. Collectively, our results suggest that ruxolitinib is potentially a safe and effective treatment for SR-cGVHD.

## Introduction

Hematopoietic stem cell transplantation (HSCT) has been one of the most important therapies for hematological malignancies. However, graft-versus-host disease (GVHD) remains an unremovable barrier, leading to late morbidity and mortality (1). Corticosteroids are the first-line treatment for GVHD. Unfortunately, more than 50% of patients with chronic GVHD (cGVHD) fail to achieve remission (2). Despite various clinical trials, no global consensus has been reached regarding second-line therapy for cGVHD (3).

Ruxolitinib, an oral JAK1/2 kinase inhibitor, was approved for intermediate-or high-risk myelofibrosis in 2011 (4) and for polycythemia vera with an inadequate response to or intolerance to hydroxyurea in 2014 (5). In addition, the JAK/STAT signaling pathway plays an important role in immune cell activation and tissue inflammation during GVHD (6, 7). Researchers have already confirmed the effect of ruxolitinib, which reduces the incidence and severity of aGVHD while preserving graft-versus-leukemia effects in preclinical models (8–10). Afterwards, ruxolitinib was subsequently reported to have shown encouraging outcomes in curing patients with aGVHD (11–14). On May 24, 2019, ruxolitinib was approved by the Food and Drug Administration (FDA) as a treatment for steroid-refractory aGVHD (SR-aGVHD) in adult and pediatric patients aged 12 years and older (15).

In 2015, Zeiser et al. first reported that ruxolitinib produced encouraging results in cGVHD therapy (16). In 2020, Zeiser et al. reported that ruxolitinib showed superior efficacy to the best available therapy (BAT) in a phase 3 trial of patients with SR-cGVHD. However, no large-scale study has focused on the efficiency and toxicity of ruxolitinib in the treatment of cGVHD among Chinese people. Here, we report a single-center retrospective study of 70 patients who received ruxolitinib as a salvage therapy for steroid-refractory cGVHD (SR-cGVHD) in our center between March 2017 and December 2019 to evaluate the safety and efficacy of ruxolitinib after HSCT.

## Methods

### Study Subjects and Data Collection

In this retrospective study, data from 70 patients who received HSCT between September 2009 and September 2019 and developed SR-cGVHD between March 2017 and December 2019 at the First Affiliated Hospital of Soochow University were collected for analysis. This study was conducted in accordance with the *Declaration of Helsinki* and approved by the ethics committee of the First Affiliated Hospital of Soochow University.

### Inclusion and Exclusion Criteria

Patients who underwent HSCT and developed SR-cGVHD at the First Affiliated Hospital of Soochow University were included in the study. When devising inclusion and exclusion criteria, the REACH3 study was used as a reference. Inclusion criteria were as follows: 1) aged > 12 years; 2) complete hematopoietic reconstitution (absolute neutrophil counts > 1.0*10^9^/L and platelet counts > 25*10^9^/L) after HSCT; and 3) a diagnosis of SR-cGVHD according to the NIH criteria (17), including no response to a minimum of 1 mg/kg/day of prednisone therapy after 1 week, as well as disease persistence without improvement after treatment with prednisone at > 0.5 mg/kg/day or 1 mg/kg/every other day for at least 4 weeks or an increase to a prednisolone dose to > 0.25 mg/kg/day after 2 unsuccessful attempts to taper the dose. The exclusion criteria were as follows: 1) relapse of underlying disease before the use of ruxolitinib for treatment, 2) uncontrolled infections or severe organ damage not related to cGVHD, and 3) enrollment in other clinical studies of cGVHD treatments at the start of the research.

### Conditioning Regimens for HSCT

The conditioning regimen for patients diagnosed with aplastic anemia (AA) was the FCA-based conditioning regimen, including IV fludarabine at 30 mg/m^2^/d on days -9 to -6, IV cyclophosphamide (CTX) at 50 mg/m^2^/d on days -5 to -2 and IV anti-thymocyte globulin (ATG) at 3.0 mg/kg^2^/d on days -5 to -2. Other patients who received HLA-matched sibling, unrelated or haploidentical transplantation were administered a Bu/Cy-based regimen consisting of oral semustine at 250 mg/m^2^/d on day -10, IV cytarabine at 4 g/m^2^/d on days -9 to -8, IV busulfan at 4 mg/kg/d from day -7 to day -5, and IV CTX at 1.8 g/m^2^/d from days -4 to -3.

### GVHD Prophylaxis

Patients who underwent HLA-matched sibling transplantation received a GVHD prophylaxis strategy consisting of cyclosporin A (CsA) and methotrexate (MTX). The GVHD prophylaxis strategy for unrelated or haploidentical transplantation patients consisted of CsA, MTX, mycophenolate mofetil (MMF) and ATG or ALG. CsA was administered at a dose of 3 mg/kg/day by continuous infusion over 24 h from day -10 until patients were able to switch to the oral formulation, with a target blood concentration ranging from 200 to 300 ng/ml. MTX was administered intravenously at a dose of 15 mg/m^2^ on day +1 and 10 mg/m^2^ on days +3, days +6 and days +11. MMF was administered at an oral dose of 250 mg twice daily from day -10 until day +30. ATG/ALG was administered intravenously at a dose of 2.5 mg/kg/d from day -5 to day -2.

### Clinical Definitions

cGVHD was diagnosed and graded according to the 2014 National Institute of Health (NIH) criteria (17). We assessed the treatment efficacy 24 weeks after the initiation of ruxolitinib therapy. Treatment responses to ruxolitinib were defined according to a previous study (16). The overall response rate (ORR) was defined as the percentage of patients assessed as achieving a complete response (CR) or partial response (PR). CR was defined as the absence of any manifestation related to cGVHD, and PR was defined as improvement in at least one specific target organ without deterioration in any other organ according to the NIH consensus (18). Events for failure-free survival (FFS) included relapse or recurrence of underlying disease or death due to underlying disease, nonrelapse mortality (NRM) and addition or initiation of another systemic therapy for cGVHD. Disease relapse was defined as morphological or cytogenetic evidence of disease with pretransplantation characteristics or morphological evidence without pretransplantation characteristics. NRM included mortality of patients who did not die due to the progression of underlying diseases.

### Laboratory Studies and Analysis of Lymphocyte Subsets

Blood samples were collected from all patients 1-3 months before and after ruxolitinib treatments, at least once per time window, for the detection of different lymphocyte subsets using flow cytometry. Blood samples were collected in EDTA anticoagulant tubes and processed within an hour for multiparameter flow cytometry analyses. Phenotyping of T cells, B cells, NK cells and other cell types was performed. Samples were stained with the following antibodies: anti-CD3, anti-CD4, anti-CD8, anti-CD19, anti-CD16, anti-CD56, anti-CD69, anti-CD25, anti-CD127, anti-CD27 and Ig-D. CD19^+^CD3^-^ cells were defined as total B cells, CD19^+^CD27^-^IgD^+^ cells were defined as naive B cells, CD19^+^CD27^+^IgD^+^ cells were defined as marginal zone B cells and CD19^+^CD27^+^IgD^-^ were defined as classical traditional B cells.

### Safety and Adverse Events

Safety was assessed by monitoring the occurrence, duration, and severity of adverse events. Adverse events were assessed according to the Common Terminology Criteria for Adverse Events, version 4.03 (https://evs.nci.nih.gov/ftp1/CTCAE/CTCAE_4.03/CTCAE_4.03_2010-06-14_QuickReference_8.5x11.pdf).

### Statistical Analysis

Our results were analyzed using SPSS 22.0 software. Normally distributed data were analyzed with Student’s t test, and nonparametric comparisons of two means were performed using the Mann-Whitney U test or the chi-square test. In the risk factor analysis, a logistic regression model was used. Time to CR, PR, NR and overall survival (OS) were defined as the time from ruxolitinib treatment to the event. Spearman’s rank correlation analysis was used. OS was analyzed using the Kaplan–Meier methodology. Comparisons were performed using the log-rank test. Cumulative incidence analysis was used to assess the incidence of relapse and NRM. A two-tailed *P* < 0.05 was considered statistically significant.

## Results

### Clinical Characteristics

A cohort of 70 patients were enrolled in this study. All patients received HSCT between September 2009 and September 2019 and developed cGVHD between March 2017 and December 2019. The detailed information is outlined in **Table 1**. The median age of the patients was 35 years (range 13-63 years). Acute myelogenous leukemia (AML) and acute lymphoblastic leukemia (ALL) were the most common underlying diseases. Matched donor transplantation was performed on 29 patients including 27 patients with related donor and 2 patients with unrelated donor, and haploidentical donor transplantation was performed on 41 patients. In this study, 32 patients received grafts of peripheral blood stem cells alone, and others received grafts combining bone marrow and peripheral blood stem cells. The median counts of transplanted mononuclear cells and CD34^+^ cells were 11.4*10^8/kg (range 3.43-29.96) and 3.80*10^6/kg (range 2.00-21.22), respectively. After HSCT, the median times of neutrophil and platelet reconstitution were 12 (range 10-23) days and 17 (range 8-80) days, respectively. The most commonly occurring complication after transplantation was bacterial infections, followed by hemorrhagic cystitis and virus infections. Forty-two patients had previously experienced acute GVHD, and 4 of them had been treated with ruxolitinib.

**Table 1:** Clinical characteristics of patients with steroid-refractory chronic graft-versus-host disease.

N (%)	
**Age (median, range)**	35 (13-63)
**Sex**	
Male	42 (60.0%)
Female	28 (40.0%)
**Diagnosis**	
Acute myeloblastic leukemia	24 (34.3%)
Acute lymphoblastic leukemia	24 (34.3%)
Chronic myeloblastic leukemia	5 (7.1%)
Chronic lymphoblastic leukemia	1 (1.4%)
Myelodysplastic syndrome	9 (12.9%)
Aplastic anemia	4 (5.7%)
Non-Hodgkin lymphoma	3 (4.3%)
**Status at HSCT**	
CR	41 (58.6%)
PR	2 (2.9%)
SD	12 (17.1%)
Others	15 (21.4%)
**Type of transplant**	
Matched donor	29 (41.4%)
Haploidentical donor	41 (58.6%)
**Graft Source**	
Peripheral blood stem cells	32 (45.7%)
Bone marrow + Peripheral blood stem cells	38 (54.3%)
**Transplanted cell count (median, range)**	
MNC (10^^8^/kg)	11.4 (3.43-29.96)
CD34 (10^^6^/kg)	3.80 (2.00-21.22)
**GVHD prophylaxis**	
CsA + MTX	29 (41.4%)
CsA + MTX + MMF	41 (58.6%)
**Days of reconstitution after HSCT (median, range)**	
NE > 1.0*10^9/L	12 (10-23)
PLT > 20*10^9/L	13 (8-80)
**Complications**	
Bacterial Infections	49 (70.0%)
Hemorrhagic cystitis	11 (15.7%)
CMV infection	10 (14.3%)
EBV infection	5 (7.1%)
**aGVHD**	
None	28 (40.0%)
Grade 1-2	22 (31.4%)
Grade 3-4	20 (28.6%)

HSCT, hematopoietic stem cell transplantation; CR, complete remission; PR, partial remission; SD, steady disease; MNC, mononuclear cell; CsA, cyclosporin A; MTX, methotrexate; MMF, mycophenolate mofetil; ATG, anti-thymocyte globulin; NE, neutrophil; PLT, platelet; CMV, cytomegalovirus; EBV, Epstein-Barr virus; aGVHD, acute graft-versus-host disease.

### cGVHD Grade and Organ Classification

The median time of cGVHD occurrence after HSCT was 317 days (range 101-3078). Twenty-three patients (32.9%) had mild cGVHD, 38 (54.3%) had moderate cGVHD, and 9 (12.8%) had severe cGVHD. Multiple organs were involved in 33 (47.1%) patients. By analyzing the targeted organs, as shown in **Table 2**, we found that the most commonly involved organ was the skin, which was affected in 28 (40.0%) patients, and the skin had the highest percentage of severe cGVHD (39.3%, 11/28). Lung, liver and gut cGVHD occurred less frequently than skin cGVHD, and severe symptoms occurred in 27.3% (6/22), 32.0% (8/25) and 30.0% (6/20) of patients, respectively. Eye cGVHD occurred in only 9 patients, and it was graded as mild or moderate. Kidney and joint cGVHD were very rarely observed in this study. In addition, skin cGVHD mostly occurred in the haploidentical HSCT group (21/41, 51.2%), while lung cGVHD was mostly common in the matched HSCT group (13/29, 44.8%). For patients who had previously been diagnosed with aGVHD, 14.3% (6/42) were graded into severe cGVHD, while the percentage of patients who had not experienced aGVHD was only 10.7% (3/28) (*P*=0.048).

**Table 2:** Characteristics of steroid-refractory chronic graft-versus-host disease.

N (%)	
**Days from transplantation to cGVHD**	
Median (range)	317 (101-3078)
**cGVHD grade at baseline**	
Mild	23 (32.9%)
Moderate	38 (54.3%)
Severe	9 (12.8%)
**Organ affected by cGVHD**	
Eye	9 (12.9%)
Mouth	6 (8.6%)
Skin	28 (40.0%)
Lung	22 (31.4%)
Liver	25 (35.7%)
Kidney	2 (2.9%)
Gut	20 (28.6%)
Joint	5 (7.1%)
**Previous lines of therapy**	
Steroids alone	19 (27.1%)
Steroids and others	51 (72.9%)

cGVHD, chronic graft-versus-host disease.

### Treatment Efficacy

**Figure 1:**
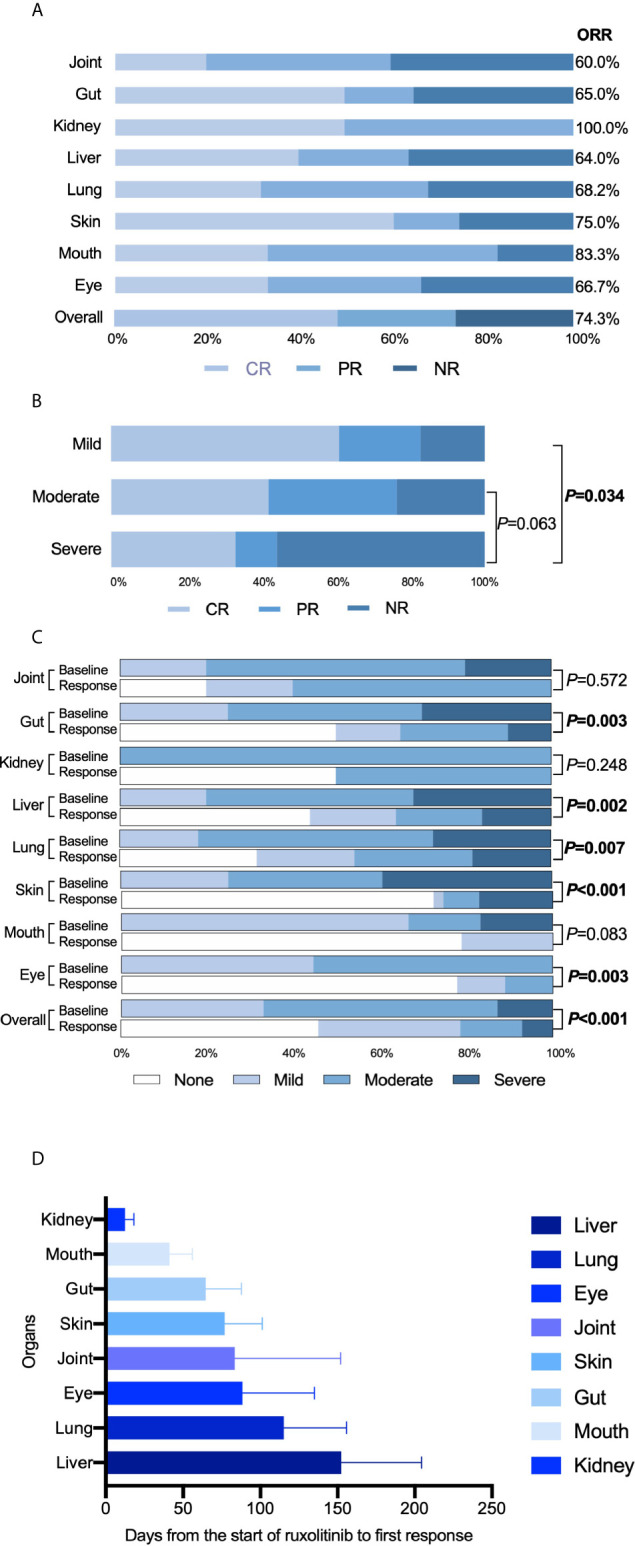
Treatment efficacy of ruxolitinib. **(A)** Response rates of different organs. **(B)** Comparison of treatment efficacy among patients with mild, moderate and severe cGVHD. **(C)** Comparison of the cGVHD grades of different organs before and 24 weeks after ruxolitinib treatments. **(D)** Time, in days, from the start of ruxolitinib administration to the response of different organs.

All patients received ruxolitinib (10-20 mg/d) as salvage therapy for cGVHD. Response rates were evaluated 24 weeks after ruxolitinib initiation. As shown in **Figure 1A**, after 24 weeks, the ORR to ruxolitinib therapy in patients with SR-cGVHD was 74.3% (52/70), including 34 patients with a CR (48.6%) and 18 with a PR (25.7%). Except for kidney and joint cGVHD cases that were too few to be analyzed, the mouth was the organ with the best response at 83.3% ORR, and the skin was the organ that achieved the highest CR of 60.7%. The ORR in patients with liver cGVHD was the lowest at only 64.0%. For patients diagnosed with different severity grades, we found that patients with severe cGVHD showed a worse ORR than patients with mild cGVHD (44.4% vs 82.6%, *P*=0.034) or moderate cGVHD (44.4% *vs* 76.3% *P*=0.063) (**Figure 1B**). After 24 weeks of treatment, we reevaluated the cGVHD severity in every patient and discovered significant reductions in the grades of cGVHD at baseline and after 24 weeks of therapy in most organs (**Figure 1C**). Next, we compared the days from ruxolitinib initiation to response among different organs, and the median time for patients with liver cGVHD to achieve remission was longer than that of other patients (125 days *vs* 49 days, *P*=0.019) (**Figure 1D**).

### Steroid and Other Combination Treatments

At the initiation of ruxolitinib treatment, all patients were receiving steroid treatments. The median dose of steroid was 1mg/kg/d (rang 0.5-2). After 4 weeks of ruxolitinib treatment, 16 patients have stopped steroid treatments and 8 patients were capable to reduce their steroid doses owing to improved symptoms. 24 weeks after ruxolitinib treatments, 18 patients have been dead, 38 patients were finally able to withdraw steroid treatments and 14 patients were still with steroids treatments with median dose of 1mg/kg/d (rang 0.5-2). In these 14 patients, 9 patients showed no response to ruxolitinib treatments and 5 patients were steroid dependent.

Besides steroid treatments, some immunosuppressor treatments were also involved. In total, 40 patients were receiving different immunosuppressor treatments at the start of ruxolitinib treatments, including tacrolimus (TAC) in 21 patients, cyclosporin A (CsA) in 12 patients and mycophenolate mofetil (MMF) in 7 patients. After 24 weeks treatments, immunosuppressors were discontinued in 31 patients and 2 patients were still receiving TAC for treatments.

### Adverse Events

Cytopenia was the most common adverse event occurring after ruxolitinib treatments (36/70, 51.4%). Anemia was the most common form, and thrombocytopenia was the second most common form. However, severe thrombocytopenia (grade III or IV) was observed in 15 of 28 patients, while severe anemia (grade III or IV) was observed only in 8 of 29 patients. Cytomegalovirus (CMV) reactivation occurred in 8 patients, while Epstein-Barr virus (EBV) and herpes infections occurred in 2 patients (**Table 3**). Viral reactivation was quickly controlled by antiviral therapy, and no other complications were observed.

**Table 3:** Adverse effects of ruxolitinib treatment on patients with steroid-refractory chronic graft- versus-host disease.

	N (%)
**Total**	50 (71.4%)
**Cytopenia**	36 (51.4%)
Anemia	29 (41.4%)
Leukopenia	21 (30.0%)
Thrombocytopenia	28 (40.0%)
**Liver function damage**	6 (8.6%)
**Kidney function damage**	1 (1.4%)
**CMV infection**	8 (11.4%)
**EBV infection**	2 (2.9%)
**Herpes virus infection**	2 (2.9%)
**TMA**	7 (10.0%)

CMV, cytomegalovirus; EBV, Epstein-Barr virus; TMA, thrombotic microangiopathy.

### Immune Function

**Figure 2:**
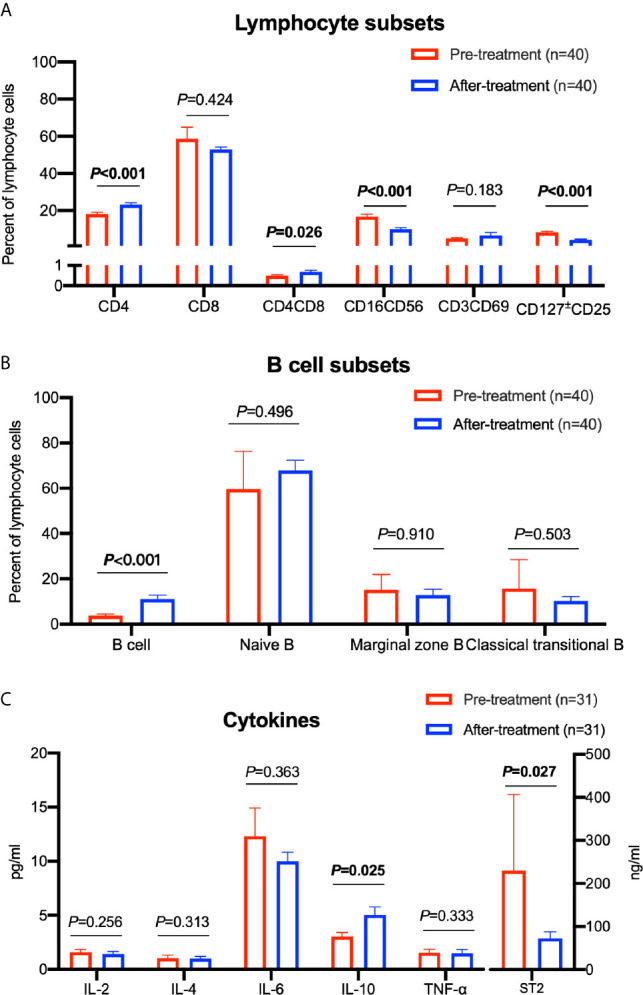
Comparison of different lymphocyte subsets and cytokine levels before and after treatment with ruxolitinib. **(A)** Comparison of different lymphocyte cell subsets. **(B)** Comparison of different B cell subsets. **(C)** Comparison of different cytokine levels. Paired sample t test was used for the analysis. The numbers of patients are indicated in each graph.

We analyzed different lymphocyte subsets during the 3 months before and after ruxolitinib treatments. The median date of the collected sample before and after ruxolitinb treatments were 54 days (range 28-88) and 63 days (range 34-94) respectively. A correlation analysis between age, lymphocyte subsets, and cytokines was performed to exclude the effect of age on different lymphocyte subsets and cytokine levels, and only naïve B cells had a negative correlation with age (**Supplementary Table S1**). CD4 lymphocytes were increased after treatment from 18.20% to 23.22% (*P*<0.001). The same trend was observed in the DP cell (CD4^+^CD8^+^) group, which increased from 0.50% to 0.68% (*P*=0.026). The numbers of both regulatory T cells (CD4^+^CD127 ^±^ CD25^+^) and NK cells (CD16^+^CD56^+^) decreased by approximately half after ruxolitinib treatment (*P*<0.001 for both) (**Figure 2A**). By analyzing the B cells of some patients, we made the novel discovery that the proportion of total B cells among lymphocytes nearly tripled from 3.69% to 11.16% (*P*<0.001). In a detailed analysis of various B cell subsets, no significant differences were observed among naïve B cells, marginal zone B cells (MZ B) and classical traditional B cells (**Figure 2B**).

In addition, we examined the levels of inflammatory cytokines in patients during treatment. We observed a significant increase in IL-10 levels from 3.02 pg/ml to 5.04 pg/ml (*P*=0.025). Moreover, we detected decreased levels of suppression of tumorigenicity 2 (ST2), a definite predictor of aGVHD, decreased by over 66% from 229.90 ng/ml to 72.65 ng/ml after ruxolitinib treatment (*P*=0.027) (**Figure 2C**).

For a more detailed analysis, we compared the variations among the skin, liver, lung and gut. In these four organs, the trends of variation in different cell subsets were basically the same (**Supplementary Figure S1**). Regarding cytokines, patients with skin cGVHD presented a significant decrease in IL-6 levels (*P*=0.008) and an increase in IL-10 levels (*P*=0.014) after ruxolitinib treatment. However, significant differences were not observed among patients with liver, lung and gut cGVHD (**Supplementary Figure S1**).

### Long-Term Outcomes

**Figure 3:**
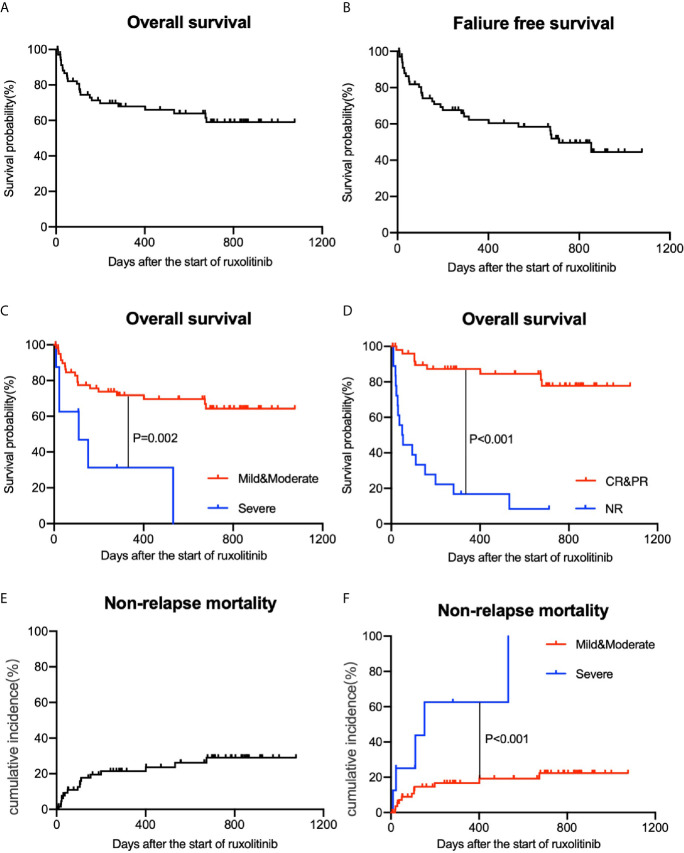
Overall survival (OS) and nonrelapse mortality (NRM) of patients with cGVHD. **(A)** OS of all patients. **(B)** Failure-free survival (FFS) of all patients. **(C)** Comparisons of OS among different grade groups. **(D)** Comparisons of OS among different treatment efficacy groups. **(E)** NRM of all patients. **(F)** Comparisons of NRM among different grade groups.

The median follow-up time of this study was 401 days (range 6-1076 days) after the initiation of ruxolitinib. The one-year estimated survival rate of the whole group was 66.0% (54.4–77.6%, 95% CI) (**Figure 3A**). The FFS estimate of the study at one year was 60.4% (48.2–72.6%, 95% CI) (**Figure 3B**). At the one-year follow-up, the estimated survival rate of patients with mild and moderate cGVHD was 69.6% (57.4–81.8%, 95% CI), which was better than that of patients with severe cGVHD (31.3%, 0.0–66.2%, 95% CI) (*P*=0.002) (**Figure 3C**). Patients who achieved CR and PR achieved better survival outcomes (84.5%, 73.9–95.1%, 95% CI) than those who showed NR to ruxolitinib treatments (16.7%, 0–34.3%, 95% CI) (*P*<0.001) (**Figure 3D**). cGVHD relapse occurred in six patients after decreases in the ruxolitinib dose or discontinuation, among which 3 patients responded to the restart of ruxolitinib therapy and achieved a response later, while the others died from cGVHD progression.

Twenty-five patients had died by the last follow-up date. Approximately half of the deaths were associated with underlying disease progression (11/25). Others included uncontrolled severe cGVHD (4/25), thrombotic microangiopathy (4/25) and complicated infections or multiple organ dysfunction syndrome (4/25). The cumulative incidence of NRM at the one-year follow-up was 20.0% (0.0-31.8%, 95% CI) (**Figure 3E**). For patients with mild and moderate cGVHD, the one-year NRM was only 16.7% (6.7-26.7%, 95% CI). However, for severe cGVHD patients, NRM at the one-year follow-up was up to 62.5% (22.7-100.0%, 95% CI) (**Figure 3F**).

## Discussion

cGVHD remains one of the major hurdles to the success of HSCT. Although corticosteroid treatment has saved millions of lives of patients with cGVHD, no consensus on second-line treatments has been established for patients with SR-cGVHD. Ruxolitinib, a JAK1/2 kinase inhibitor, was first reported in a 2015 multicenter retrospective survey by Zeiser et al. (16) to have exerted satisfactory therapeutic effects on SR-cGVHD, supported by a favorable ORR of 85.4%. Among other commonly used second line cGVHD treatments, extracorporeal photopheresis (ECP) treatments were reported to achieve an ORR of 56.0% in a randomized controlled study (19) and an ORR of 67.0% in a retrospective multicenter study (20) of patients with cGVHD. In a large retrospective study including 269 patients with SR-cGVHD by Axt et al. (21), the ORRs of calcineurin inhibitors, MMF, mTOR inhibitors and ECP were all lower than 60.0%. Ibrutinib, a Bruton tyrosine kinase inhibitor, showed a 67.0% ORR for patients with cGVHD in a multicenter, open-label study (22). Some researchers recruited only patients with moderate and severe cGVHD into study, while many studies included patients with mild to severe cGVHD (20, 21, 23–25). In our single-center retrospective survey conducted among 70 patients diagnosed with mild, moderate and severe cGVHD, the median follow-up time was 401 (range 6-1076) days. Up to the final follow-up time, 74.3% of patients had responded to ruxolitinib, of whom 48.6% and 25.7% achieved CR and PR, respectively. A comparable ORR was reported in studies by Abedin et al. (26), Modi et al. (24) and Khoury et al. (27). Many investigators also evaluated the ORR of ruxolitinib at different time points. Abedin et al. (26) assessed the treatment efficacy at 28 days after the use of ruxolitinib; nevertheless, the ORR was only 63%. In the investigation of Modi et al. (24), treatment efficacies were evaluated at two time points. After six months of ruxolitinib therapy, the authors observed a CR in 10% of patients and PR in 37% of patients, while after 12 months, the results differed only slightly, with a CR observed in 13% of patients and PR in 30% of patients. In 2020, Zeiser et al. reported their findings from the phase 3 randomized REACH3 study of ruxolitinib compared with BAT in patients with SR-cGVHD. Ruxolitinib resulted in a significantly higher ORR at week 24 than BAT (49.7% *vs* 25.6%, *P*<0.0001), and it was the first agent to show superior efficacy to BAT in a phase 3 trial of patients with SR-cGVHD.

In the present study, mouth cGVHD had the highest ORR to ruxolitinib therapy, and skin cGVHD had the highest CR, a comparable result to the research conducted by Hurabielle et al. (28), who focused on sclerodermatous cGVHD independently. Moreover, in most studies, the mouth and skin were always the best-responding organs. The liver and lung were reported to be the organs with the worst response to ruxolitinib therapy (28–30). Additionally, the ORR in the gut, liver and lung was the lowest, and patients with liver and lung cGVHD had the longest response times in this study. Moreover, Moiseev et al. (29) and Streiler et al. (31) both reported that ruxolitinib significantly improved the respiratory function of patients with cGVHD, reduced steroid requirements and stabilized lung function in patients with bronchiolitis obliterans as a manifestation of cGVHD.

The safety of ruxolitinib treatment was also important. Hemocytopenia was the most common adverse event observed in this study of patients with cGVHD, consistent with previously reported data. In addition, Moiseev et al. (29) claimed that the severity of neutropenia and thrombocytopenia was affected by CMV reactivation (*P*=0.07), treatment with ganciclovir (*P*=0.0006), and a higher initial steroid dose (*P*=0.0017). González Vicent et al. (25) also determined that the incidence of neutropenia was related to the appearance of CMV and treatment with ganciclovir. In the majority of published articles, the incidence of CMV activation was reported to be greater than 10% (32, 33). However, in the present study, a low risk of reactivating CMV, EBV or herpes virus infections was observed, and reactivation was quickly controlled by antiviral therapies. Additionally, liver and kidney toxicities were uncommon in all published articles, including articles published by our group (24, 28, 29, 34). One possible reason for the low occurrence of adverse effects in this study might be the relatively low dose of ruxolitinib.

As reported before, the JAK1/2 inhibitor ruxolitinib influences the immune response after HSCT (6, 7, 10). In preclinical research, ruxolitinib has been reported to reverse dysregulated T helper cell responses and control autoimmunity resulting from signal transducer and activator of transcription 1 (STAT1) gain-of-function mutations (35). Vicent et al. (25) discussed the variations in the immune system before and after patients with cGVHD received ruxolitinib treatments, in which ruxolitinib was associated with increased numbers of CD4^+^ T cells and B cells and decreased numbers of NK cells and CD4^+^ Tregs. Notably, we observed increased numbers of CD4^+^ and CD8^+^ DP cells after ruxolitinib treatments. DP cells are a well-described T cell developmental stage within the thymus; in patients with cGVHD, a higher percentage of DP cells indicates better thymus function and less GVHD damage (36, 37). B cells play an indispensable role in the occurrence and development of cGVHD (38, 39); however, few researchers have analyzed the changes in specific B cell subsets before and after ruxolitinib treatment. Studies from both McManigle (40) and Yehudai-Ofir (41) reported that CD27 is normally expressed on B cells and that CD27-positive B cells are proportionally increased in patients with cGVHD. In the present study, the percentage of CD27-negative naïve B cells increased, while the percentages of MZ B cells and classical traditional B cells, which were both CD27-positive, decreased after treatment. Among cytokines, we detected an increase in the levels of IL-10, a definite inhibitory mediator of GVHD (42), after ruxolitinib treatments. In further analyses, the level of the proinflammatory factor IL-6 was decreased in patients with skin cGVHD after ruxolitinib treatments, consistent with published data (43, 44). However, these variations were not observed in patients with liver, lung and gut cGVHD, whose ORRs were lower than patients with skin cGVHD.

Additionally, ST2 has been previously reported to be a specific indicator of aGVHD (45, 46). In 2015, Reichenbach et al. (47) analyzed animal GVHD models and reported that ST2 was upregulated on murine alloreactive T cells and that ST2 levels increased as experimental GVHD progressed. Compared with wild-type (WT) donor T cells, ST2^−/−^ donor T cells displayed a marked reduction in GVHD lethality. In our study, ST2 expression also fluctuated with the severity of cGVHD.

Notably, the median follow-up time in our study was 401 (range 6-1076) days, the one-year estimated survival rate was 66.0% (54.4–77.6%, 95% CI), and the one-year estimated FFS rate was 60.4% (48.2–72.6%, 95% CI). In our study, patients with severe cGVHD experienced a significantly shorter OS and higher NRM than patients with mild and moderate diseases. The OS of patients with mild and moderate cGVHD was 69.6%, probably because approximately two-thirds of these patients had moderate cGVHD. Considering the relatively long follow-up time compared with the studies by Zeiser et al. (16) and Moiseev et al. (27), we propose that our study describes an encouraging survival benefit for patients with SR-cGVHD.

Several limitations also existed in our study. Besides the retrospective nature of this study, it was also difficult to properly account for the effects of concurrent immunosuppressive therapies including corticosteroids and calcineurin inhibitors on the clinical course of cGVHD in addition to the effect of ruxolitinib.

Interestingly, in addition to salvage therapy for SR-cGVHD, ruxolitinib showed excellent performance as a prophylactic agent for GVHD in place of calcineurin inhibitors. Kröger et al. (48) reported on 12 patients who used ruxolitinib during the peritransplantation period. The incidence of grade II–IV aGVHD on day +100 was only 8%, and no NRM was recorded. In the study designed by Zhao et al. (49), after the replacement of a calcineurin inhibitor with ruxolitinib once patients showed intolerance or contraindication to CsA or TAC, only two of ten patients developed aGVHD, and 3 patients developed cGVHD after tapering or stopping ruxolitinib. Moreover, in July 2020, Saraceni et al. (50) reported that patients with cGVHD who were diagnosed with severe coronavirus disease 2019 (COVID-19) were successfully treated with ruxolitinib.

Collectively, the results of this study support ruxolitinib as a safe and effective option as a second-line treatment for patients with SR-cGVHD, with a high ORR of 73.4% and impressive outcomes. Further multicenter studies enrolling a larger number of participants should be conducted in the future.

## Data Availability Statement

The raw data supporting the conclusions of this article will be made available by the authors, without undue reservation.

## Ethics Statement

The studies involving human participants were reviewed and approved by ethical committee of the First Affiliated Hospital of Soochow University. Written informed consent to participate in this study was provided by the participants’ legal guardian/next of kin. Written informed consent was obtained from the individual(s), and minor(s)’ legal guardian/next of kin, for the publication of any potentially identifiable images or data included in this article.

## Author Contributions

DWu and YX designed the study. XM, QC, and ZL contributed to the collection of data. DWang analyzed the data. DWang, YL, and XL discussed and interpreted the results. DWang wrote the manuscript. All authors contributed to the article and approved the submitted version.

## Funding

This work was supported in part by grants from the National Natural Science Foundation of China (81730003, 81870120, 81800176, and 82070187), the National Key R&D Program of China (2019YFC0840604, 2017YFA0104502, and 2017ZX09304021), the Natural Science Foundation of Jiangsu Province (BK20171205 and BK20180200), the Social Development of Jiangsu Provinces (BE2019655), the Jiangsu Province Key R&D Program (BE2019798), and the Priority Academic Program Development of Jiangsu Higher Education Institutions (PAPD).

## Conflict of Interest

The authors declare that the research was conducted in the absence of any commercial or financial relationships that could be construed as a potential conflict of interest.

## Supplementary Material

Supplementary Figure 1 Comparison of different lymphocyte subsets and cytokine levels in different organs before and after treatment with ruxolitinib. **(A)** Comparison of different lymphocyte cell subsets in different organs. **(B)** Comparison of different B cell subsets in different organs. **(C)** Comparison of different cytokine levels in different organs. Paired sample t test was used for the analysis. The numbers of patients are indicated in each graph.
