# Supplementary figures and images for: Efficiency and Toxicity of Ruxolitinib as a Salvage Treatment for Steroid-Refractory Chronic Graft-Versus-Host Disease
Source: Front Immunol. 2021 Jun 30;12:673636. doi: 10.3389/fimmu.2021.673636 (PMC8278571; doi:10.3389/fimmu.2021.673636)

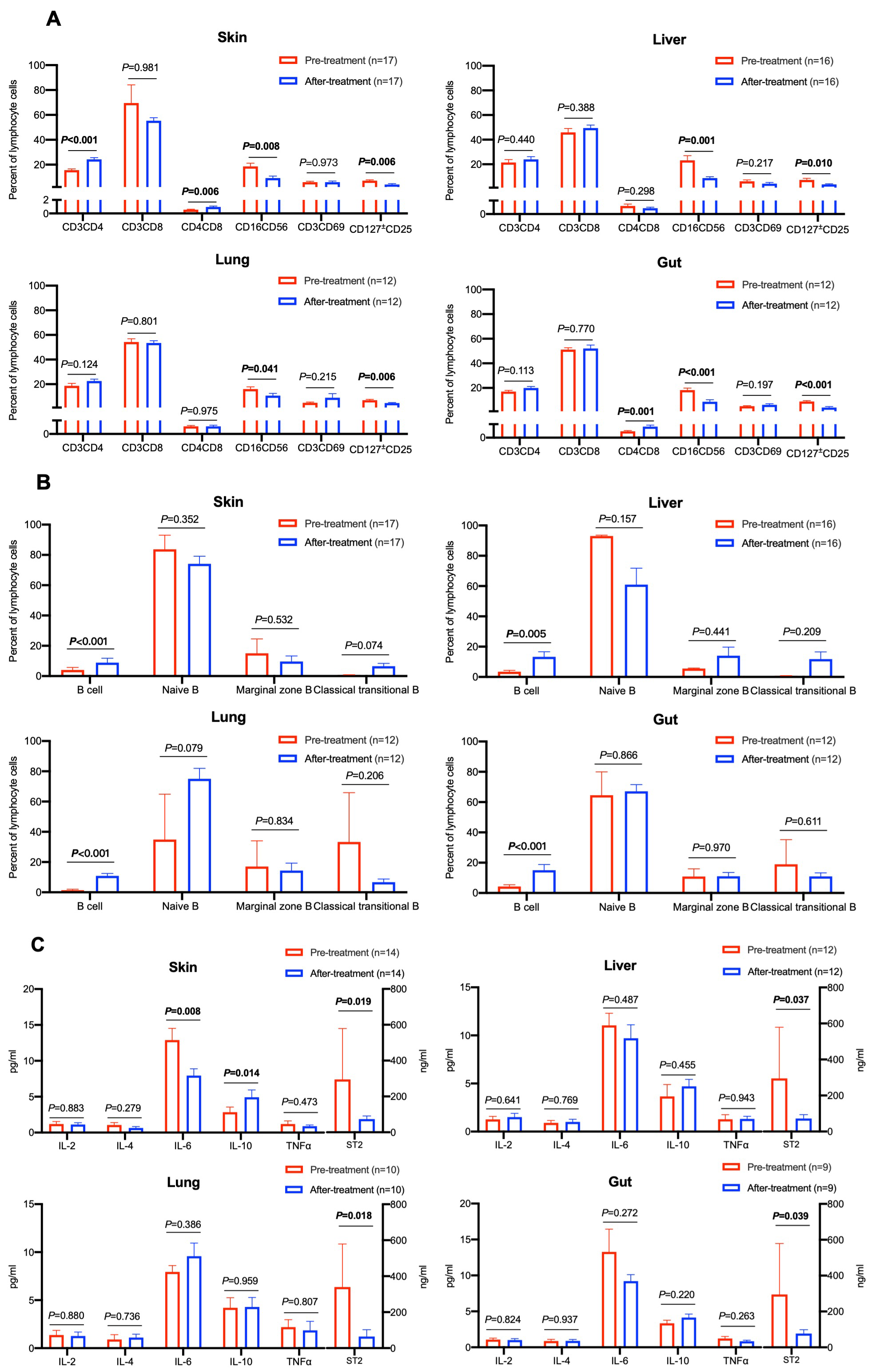

Supplement: Supplementary Figure 1 — Comparison of different lymphocyte subsets and cytokine levels in different organs before and after treatment with ruxolitinib. (A) Comparison of different lymphocyte cell subsets in different organs. (B) Comparison of different B cell subsets in different organs. (C) Comparison of different cytokine levels in different organs. Paired sample t test was used for the analysis. The numbers of patients are indicated in each graph. [file Image_1.tif]
