# Supplementary material for: Efficiency and Toxicity of Ruxolitinib as a Salvage Treatment for Steroid-Refractory Chronic Graft-Versus-Host Disease
Source: Front Immunol. 2021 Jun 30;12:673636. doi: 10.3389/fimmu.2021.673636 (PMC8278571; doi:10.3389/fimmu.2021.673636)
Supplement: Supplementary file 2 [file Table_1.docx]

**TABLE S1 |** Correlation analysis of age and different immune cells and cytokine

|  | ***P* value** | ***R* value** |
| --- | --- | --- |
| **CD4** | 0.326 | -0.074 |
| **CD8** | 0.187 | 0.100 |
| **CD4CD8** | 0.414 | -0.070 |
| **CD16CD56** | 0.294 | -0.082 |
| **CD3CD69** | 0.463 | 0.061 |
| **CD127^±^CD25** | 0.060 | 0.162 |
| **B cell** | 0.680 | 0.031 |
| **Naïve B** | 0.036 | -0.422 |
| **Marginal zone B** | 0.792 | 0.056 |
| **Classical transitional B** | 0.130 | 0.312 |
| **IL-2** | 0.199 | 0.016 |
| **IL-4** | 0.767 | 0.000 |
| **IL-6** | 0.444 | 0.006 |
| **IL-10** | 0.638 | 0.002 |
| **TNF-**$\boldsymbol{\alpha}$ | 0.195 | 0.017 |
| **ST2** | 0.829 | 0.002 |

**Abbreviations:** IL-2, interleukin-2; IL-4, interleukin-4; IL-6, interleukin-6; IL-10, interleukin-10; TNF-α, tumor necrosis factor-α; ST2, suppression of tumorigenicity 2
